# 4-(3-Ammonio­prop­yl)morpholin-4-ium tetra­chloridozincate(II)

**DOI:** 10.1107/S1600536809004346

**Published:** 2009-02-18

**Authors:** Meher El Glaoui, Erwann Jeanneau, Mohamed Rzaigui, Cherif Ben Nasr

**Affiliations:** a Laboratoire de Chimie des Matériaux, Faculté des Sciences de Bizerte, 7021 Zarzouna, Tunisia; b Universtié Lyon1, Centre de Diffractométrie Henri Longchambon, 43 Boulevard du 11 Novembre 1918, 69622 Villeurbanne Cedex, France

## Abstract

In the title compound, (C_7_H_18_N_2_O)[ZnCl_4_], the Zn^II^ ion is coordinated by four Cl atoms in a close to tetra­hedral geometry. The crystal packing exhibits C—H⋯Cl, N—H⋯Cl and N—H⋯O hydrogen bonds.

## Related literature

For common applications of this material, see: Bringley & Rajeswaran (2006 ▶); Tao *et al.* (2003 ▶). For structure cohesion, see: Brammer *et al.* (2002 ▶). For a discussion of Zn—Cl distances and Cl—Zn—Cl bond angles, see: Guo *et al.* (2007 ▶); Valkonen *et al.* (2006 ▶). For computational details, see: Prince (1982 ▶); Watkin (1994 ▶).
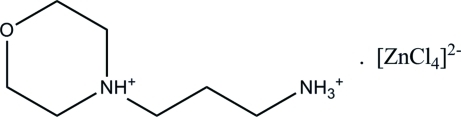


## Experimental

### 

#### Crystal data

(C_7_H_18_N_2_O)[ZnCl_4_]
*M* *_r_* = 353.42
Monoclinic, 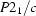

*a* = 6.2765 (2) Å
*b* = 14.3552 (4) Å
*c* = 15.4858 (6) Å
β = 100.759 (4)°
*V* = 1370.75 (8) Å^3^
*Z* = 4
Mo *K*α radiation
μ = 2.55 mm^−1^
*T* = 293 K
0.17 × 0.09 × 0.08 mm

#### Data collection

Oxford Diffraction Xcalibur area-detector diffractometer
Absorption correction: multi-scan (*CrysAlis RED*; Oxford Diffraction, 2002 ▶) *T*_min_ = 0.63, *T*_max_ = 0.82
13120 measured reflections
3304 independent reflections
2815 reflections with *I* > 2σ(*I*)
*R*_int_ = 0.021

#### Refinement

*R*[*F* ^2^ > 2σ(*F* ^2^)] = 0.019
*wR*(*F* ^2^) = 0.020
*S* = 1.04
2696 reflections
137 parameters
H-atom parameters constrained
Δρ_max_ = 0.27 e Å^−3^
Δρ_min_ = −0.19 e Å^−3^

### 

Data collection: *CrysAlis CCD* (Oxford Diffraction, 2002 ▶); cell refinement: *CrysAlis RED* (Oxford Diffraction, 2002 ▶); data reduction: *CrysAlis RED*; program(s) used to solve structure: *SIR97* (Altomare *et al.*, 1999 ▶); program(s) used to refine structure: *CRYSTALS* (Betteridge *et al.*, 2003 ▶); molecular graphics: *CAMERON* (Watkin *et al.*, 1996 ▶); software used to prepare material for publication: *CRYSTALS*.

## Supplementary Material

Crystal structure: contains datablocks global, I. DOI: 10.1107/S1600536809004346/lx2089sup1.cif

Structure factors: contains datablocks I. DOI: 10.1107/S1600536809004346/lx2089Isup2.hkl

Additional supplementary materials:  crystallographic information; 3D view; checkCIF report

## Figures and Tables

**Table 1:** Hydrogen-bond geometry (Å, °)

*D*—H⋯*A*	*D*—H	H⋯*A*	*D*⋯*A*	*D*—H⋯*A*
N2—H2⋯O^i^	0.87	1.95	2.821 (2)	173
N2—H3⋯Cl2	0.87	2.43	3.209 (2)	150
C1—H6⋯Cl2^ii^	0.96	2.72	3.653 (2)	164
C7—H18⋯Cl2^iii^	0.95	2.70	3.644 (2)	173
C5—H14⋯Cl4^ii^	0.98	2.82	3.657 (2)	144
N2—H4⋯Cl3^iii^	0.87	2.54	3.320 (2)	149
N1—H1⋯Cl3^iv^	0.88	2.42	3.206 (2)	149
C2—H7⋯Cl1^iv^	0.97	2.74	3.677 (2)	165

Symmetry codes: (i) 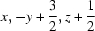; (ii) 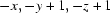; (iii) 
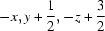
; (iv) 
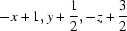
.

**Fig. 1.**
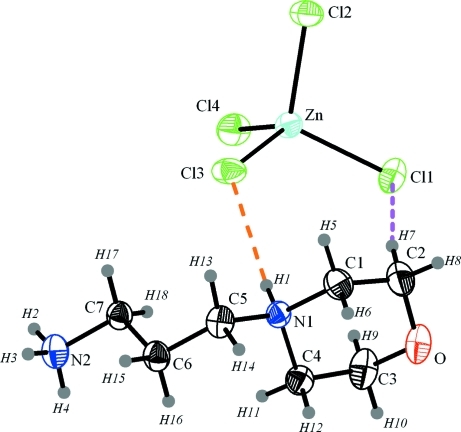
The molecular structure of the title compound, showing displacement ellipsoids drawn at the 40% probability level.

**Fig. 2.**
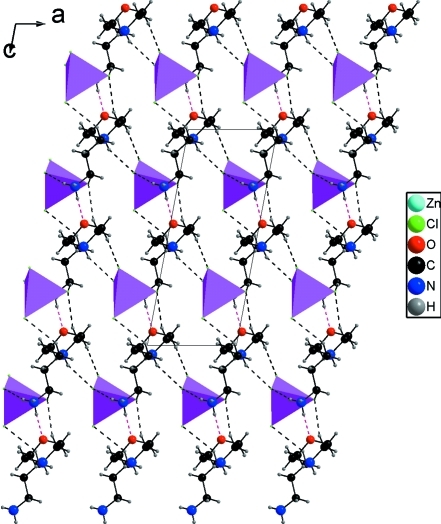
Crystal structure of (I) viewed along b axis showing the layered organization.

**Fig. 3.**
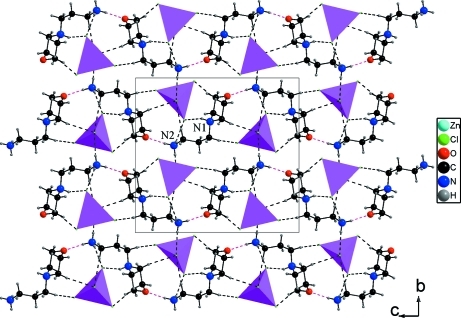
The packing of (I) viewed down the a axis showing layers at y = 1/4 and y = 3/4.

## supplementary crystallographic information

### Comment

Hybrid compounds have many practical and potential applications in various field (Tao *et al.*, 2003; Bringley and Rajeswaran, 2006). In these materials, the crystal packing is ensured by hydrogen bonds and coulombic interactions (Brammer *et al.*, 2002). Here we report the crystal structure of the title compound, 4-(3-ammoniopropyl)morpholin-4-ium tetrachlorozincate (II) (Fig. 1).

As shown in Fig. 1, to ensure charge balance, the organic species is double protonated at N1 and N2 nitrogen atoms. The structure consists essentially of an 4-(3-ammoniopropyl)morpholin-4-ium and [ZnCl_4_]^2-^ anion which are held together by N—H···Cl and C—H···Cl hydrogen bonds so as to build layers developing parallel to (a, c) planes (Fig. 2). These layers, situated at *y* = ^1^/_4_ and *y* = ^3^/_4_, are themselves interconnected by a set of N2—H···Cl hydrogen bonds (Table 1), alternating with layers, to form a three dimensional infinite network (Fig. 3). The Zn (II) ion is in tetrahedral coordination environment composed of four chloride ions. Each ZnCl4^2-^ anion is connected to its neighbors organic cations, which are associated *via* N—H···O hydrogen bonds, by N—H···Cl and C—H···Cl interactions involving four chlorine atoms (Table 1). The Cl1 and Cl4 are simple acceptors, the Cl3 is double acceptor and the Cl2 is triple acceptor of hydrogen bonds. The (N)—H···Cl distances, varying between 2.42 and 2.54 Å, are smaller than the sum of the Van der Walls radii of the chlorine and hydrogen atoms [*r*(Cl) + r(H) = 2.81 Å]. Consequently, these values correspond well to strong hydrogen bonds.

However, it is worth noticing that the Zn—Cl bond lengths and Cl—Zn—Cl bond angles in the [ZnCl_4_]^2-^ anion are not equal to one another but vary with the environment around the Cl atoms(Valkonen *et al.*, 2006). In the title compound, the Zn—Cl bond lengths vary between 2.2486 (4) and 2.2950 (4) Å. The Cl—Zn—Cl bond angles range from 104.32 (1) to 114.43 (2) °. These values indicate that the anionic [ZnCl_4_]^2-^ tetrahedron is slightly distorted (Guo *et al.*, 2007).

### Experimental

ZnCl_2_, aqueous 1*M* HCl solution and 3-Morpholinopropylamine in a 1:2:1 molar ratio were mixed and dissolved in sufficient ethanol. Single crystals suitable for X-ray diffraction were prepared by evaporation of a solution of the title compound in ethanol at room temperature after a few days.

### Refinement

The H atoms were all located in a difference map, but those attached to carbon atoms were repositioned geometrically. The H atoms were initially refined with soft restraints on the bond lengths and angles to regularize their geometry (C—H in the range 0.93–0.98, N—H in the range 0.86–0.89 and O—H = 0.82 Å) and *U*_iso_(H) (in the range 1.2–1.5 times *U*_eq_ of the parent atom), after which the positions were refined with riding constraints.

### Crystal data

**Table:**

(C_7_H_18_N_2_O)[ZnCl_4_]	*F*(000) = 720
*M**_r_* = 353.42	*D*_x_ = 1.712 Mg m^−^^3^
Monoclinic, *P*2_1_/*c*	Mo *K*α radiation, λ = 0.7107 Å
Hall symbol: -P 2ybc	Cell parameters from 7336 reflections
*a* = 6.2765 (2) Å	θ = 2.8–29.2°
*b* = 14.3552 (4) Å	µ = 2.55 mm^−^^1^
*c* = 15.4858 (6) Å	*T* = 293 K
β = 100.759 (4)°	Block, colorless
*V* = 1370.75 (8) Å^3^	0.17 × 0.09 × 0.08 mm
*Z* = 4	

### Data collection

**Table:**

Oxford Diffraction XCALIBUR area-detector diffractometer	3304 independent reflections
Radiation source: Enhance (Mo) X-ray Source	2815 reflections with *I* > 2σ(*I*)
graphite	*R*_int_ = 0.021
Detector resolution: 15.9897 pixels mm^-1^	θ_max_ = 29.3°, θ_min_ = 2.8°
φ and ω scans	*h* = −8→8
Absorption correction: multi-scan (*CrysAlis RED*; Oxford Diffraction, 2002)	*k* = −18→18
*T*_min_ = 0.63, *T*_max_ = 0.82	*l* = −18→20
13120 measured reflections	

### Refinement

**Table:**

Refinement on *F*	Hydrogen site location: inferred from neighbouring sites
Least-squares matrix: full	H-atom parameters constrained
*R*[*F*^2^ > 2σ(*F*^2^)] = 0.019	Method, part 1, Chebychev polynomial, (Watkin, 1994, *P*rince, 1982) [*w*eight] = 1.0/[A_0_*T_0_(x) + A_1_*T_1_(x) ··· + A_n-1_]*T_n-1_(x)] where A_i_ are the Chebychev coefficients listed belo*w* and x = *F* /*F*max Method = Robust Weighting (*P*rince, 1982) W = [*w*eight] * [1-(delta*F*/6*sigma*F*)^2^]^2^ A_i_ are: 8.69 -6.08 5.75
*wR*(*F*^2^) = 0.020	(Δ/σ)_max_ = 0.001
*S* = 1.04	Δρ_max_ = 0.27 e Å^−^^3^
2696 reflections	Δρ_min_ = −0.19 e Å^−^^3^
137 parameters	Extinction correction: Larson (1970), Equation 22
0 restraints	Extinction coefficient: 64 (4)
Primary atom site location: structure-invariant direct methods	

### Special details

**Table:**

Refinement. Data with I<3σ(I) were excluded from the refinement.

### Fractional atomic coordinates and isotropic or equivalent isotropic displacement parameters (Å^2^)

**Table:**

	*x*	*y*	*z*	*U*_iso_*/*U*_eq_	
Zn1	0.37044 (3)	0.371470 (11)	0.756164 (11)	0.0273	
Cl1	0.48665 (7)	0.47971 (3)	0.85946 (3)	0.0402	
Cl2	0.00747 (6)	0.35230 (3)	0.74949 (3)	0.0367	
Cl3	0.51658 (6)	0.22854 (2)	0.79921 (3)	0.0372	
Cl4	0.43433 (6)	0.42069 (3)	0.62551 (2)	0.0402	
C1	0.2040 (3)	0.73398 (12)	0.47182 (11)	0.0383	
C2	0.2358 (3)	0.83554 (14)	0.45739 (12)	0.0468	
C3	−0.0795 (3)	0.87396 (11)	0.50847 (11)	0.0441	
C4	−0.1308 (2)	0.77313 (10)	0.52356 (10)	0.0319	
C5	0.0385 (3)	0.61461 (10)	0.54896 (10)	0.0319	
C6	−0.1030 (2)	0.58498 (10)	0.61278 (9)	0.0310	
C7	−0.0059 (2)	0.60272 (10)	0.70780 (9)	0.0284	
O	0.0323 (2)	0.88265 (8)	0.43720 (8)	0.0456	
N1	0.07386 (18)	0.71776 (8)	0.54268 (7)	0.0256	
N2	−0.1501 (2)	0.56344 (9)	0.76395 (8)	0.0355	
H1	0.1513	0.7371	0.5924	0.0370*	
H2	−0.0977	0.5758	0.8191	0.0530*	
H3	−0.1610	0.5037	0.7569	0.0543*	
H4	−0.2786	0.5881	0.7508	0.0540*	
H5	0.3391	0.7034	0.4902	0.0478*	
H6	0.1254	0.7059	0.4190	0.0461*	
H7	0.3247	0.8625	0.5090	0.0563*	
H8	0.3080	0.8410	0.4080	0.0566*	
H9	0.0095	0.9001	0.5612	0.0534*	
H10	−0.2154	0.9071	0.4938	0.0536*	
H11	−0.2037	0.7673	0.5727	0.0386*	
H12	−0.2185	0.7468	0.4714	0.0373*	
H13	0.1820	0.5879	0.5638	0.0378*	
H14	−0.0305	0.5963	0.4896	0.0376*	
H15	−0.1224	0.5188	0.6059	0.0375*	
H16	−0.2428	0.6161	0.5982	0.0365*	
H17	0.1334	0.5740	0.7240	0.0348*	
H18	0.0068	0.6679	0.7193	0.0354*	

### Atomic displacement parameters (Å^2^)

**Table:**

	*U*^11^	*U*^22^	*U*^33^	*U*^12^	*U*^13^	*U*^23^
Zn1	0.02689 (9)	0.02429 (9)	0.02946 (9)	−0.00028 (6)	0.00203 (6)	0.00055 (6)
Cl1	0.0496 (2)	0.03524 (19)	0.03582 (19)	−0.00756 (15)	0.00812 (16)	−0.00963 (14)
Cl2	0.02569 (16)	0.03157 (17)	0.0513 (2)	−0.00010 (12)	0.00342 (14)	0.00177 (15)
Cl3	0.02699 (16)	0.02632 (16)	0.0545 (2)	0.00182 (12)	−0.00201 (15)	0.00397 (15)
Cl4	0.0402 (2)	0.0493 (2)	0.03073 (18)	−0.00006 (16)	0.00604 (15)	0.00497 (15)
C1	0.0350 (8)	0.0496 (9)	0.0336 (8)	0.0016 (7)	0.0147 (6)	0.0046 (7)
C2	0.0456 (9)	0.0538 (10)	0.0415 (9)	−0.0109 (8)	0.0096 (7)	0.0126 (8)
C3	0.0648 (11)	0.0334 (8)	0.0370 (8)	0.0071 (7)	0.0177 (8)	0.0041 (6)
C4	0.0333 (7)	0.0331 (7)	0.0306 (7)	0.0031 (6)	0.0093 (6)	0.0007 (5)
C5	0.0405 (8)	0.0261 (7)	0.0297 (7)	0.0009 (5)	0.0084 (6)	−0.0024 (5)
C6	0.0367 (7)	0.0256 (7)	0.0300 (7)	−0.0060 (5)	0.0047 (6)	−0.0010 (5)
C7	0.0318 (7)	0.0238 (6)	0.0293 (7)	−0.0020 (5)	0.0052 (5)	0.0007 (5)
O	0.0610 (8)	0.0437 (7)	0.0337 (6)	0.0007 (5)	0.0133 (5)	0.0131 (5)
N1	0.0283 (6)	0.0286 (6)	0.0192 (5)	−0.0033 (4)	0.0027 (4)	−0.0002 (4)
N2	0.0441 (7)	0.0331 (6)	0.0310 (6)	−0.0010 (5)	0.0119 (5)	0.0018 (5)

### Geometric parameters (Å, °)

**Table:**

Zn1—Cl1	2.2515 (4)	C5—H13	0.966
Zn1—Cl2	2.2779 (4)	C5—H14	0.976
Zn1—Cl3	2.2950 (4)	C6—C7	1.5056 (19)
Zn1—Cl4	2.2486 (4)	C6—H16	0.973
O—C2	1.427 (2)	C6—H15	0.961
O—C3	1.419 (2)	C7—N2	1.4790 (18)
C2—C1	1.494 (2)	C7—H18	0.954
C2—H7	0.966	C7—H17	0.957
C2—H8	0.962	N2—H2	0.875
C1—N1	1.5036 (18)	N2—H3	0.866
C1—H5	0.950	N2—H4	0.869
C1—H6	0.961	C4—C3	1.510 (2)
N1—C5	1.5032 (17)	C4—H11	0.963
N1—C4	1.4922 (18)	C4—H12	0.965
N1—H1	0.875	C3—H9	0.974
C5—C6	1.508 (2)	C3—H10	0.966
			
Cl1—Zn1—Cl2	107.710 (16)	C5—C6—C7	114.36 (12)
Cl1—Zn1—Cl3	110.593 (17)	C5—C6—H16	109.5
Cl2—Zn1—Cl3	104.316 (14)	C7—C6—H16	109.4
Cl1—Zn1—Cl4	109.501 (17)	C5—C6—H15	106.5
Cl2—Zn1—Cl4	109.997 (17)	C7—C6—H15	107.2
Cl3—Zn1—Cl4	114.428 (17)	H16—C6—H15	109.8
C2—O—C3	109.87 (13)	C6—C7—N2	109.25 (12)
O—C2—C1	110.87 (14)	C6—C7—H18	110.7
O—C2—H7	110.3	N2—C7—H18	107.7
C1—C2—H7	109.9	C6—C7—H17	111.5
O—C2—H8	108.8	N2—C7—H17	108.1
C1—C2—H8	107.1	H18—C7—H17	109.5
H7—C2—H8	109.9	C7—N2—H2	109.7
C2—C1—N1	111.48 (13)	C7—N2—H3	110.2
C2—C1—H5	111.0	H2—N2—H3	109.4
N1—C1—H5	106.6	C7—N2—H4	110.5
C2—C1—H6	110.1	H2—N2—H4	108.0
N1—C1—H6	107.0	H3—N2—H4	109.0
H5—C1—H6	110.5	N1—C4—C3	109.92 (13)
C1—N1—C5	107.86 (11)	N1—C4—H11	108.4
C1—N1—C4	109.72 (11)	C3—C4—H11	110.6
C5—N1—C4	113.91 (11)	N1—C4—H12	107.1
C1—N1—H1	107.7	C3—C4—H12	110.5
C5—N1—H1	108.7	H11—C4—H12	110.3
C4—N1—H1	108.8	C4—C3—O	110.79 (13)
N1—C5—C6	115.63 (11)	C4—C3—H9	110.1
N1—C5—H13	105.3	O—C3—H9	109.2
C6—C5—H13	111.7	C4—C3—H10	107.7
N1—C5—H14	104.5	O—C3—H10	108.4
C6—C5—H14	109.2	H9—C3—H10	110.5
H13—C5—H14	110.3		

### Hydrogen-bond geometry (Å, °)

**Table:**

*D*—H···*A*	*D*—H	H···*A*	*D*···*A*	*D*—H···*A*
N2—H2···O^i^	0.87	1.95	2.821 (2)	173
N2—H3···Cl2	0.87	2.43	3.209 (2)	150
C1—H6···Cl2^ii^	0.96	2.72	3.653 (2)	164
C7—H18···Cl2^iii^	0.95	2.70	3.644 (2)	173
C5—H14···Cl4^ii^	0.98	2.82	3.657 (2)	144
N2—H4···Cl3^iii^	0.87	2.54	3.320 (2)	149
N1—H1···Cl3^iv^	0.88	2.42	3.206 (2)	149
C2—H7···Cl1^iv^	0.97	2.74	3.677 (2)	165

Symmetry codes: (i) *x*, −*y*+3/2, *z*+1/2; (ii) −*x*, −*y*+1, −*z*+1; (iii) −*x*, *y*+1/2, −*z*+3/2; (iv) −*x*+1, *y*+1/2, −*z*+3/2.
